# Peripheral Microangiopathy Changes in Pulmonary Arterial Hypertension Related to Systemic Sclerosis: Data From a Multicenter Observational Study

**DOI:** 10.3389/fcvm.2022.924899

**Published:** 2022-07-11

**Authors:** Dilia Giuggioli, Valeria Riccieri, Edoardo Cipolletta, Nicoletta Del Papa, Francesca Ingegnoli, Amelia Spinella, Greta Pellegrino, Anna Maria Risa, Marco de Pinto, Silvia Papa, Giuseppe Armentaro, Rossella De Angelis

**Affiliations:** ^1^Scleroderma Unit, Rheumatology Unit, University Hospital of Modena and Reggio Emilia, Modena, Italy; ^2^Scleroderma Clinic, Rheumatology Unit, Sapienza University of Rome, Rome, Italy; ^3^Rheumatology Unit, Department of Clinical and Molecular Sciences, Polytechnic University of Marche, Ancona, Italy; ^4^Clinical Rheumatology Unit, ASST Pini-CTO, Department of Clinical Science and Community Health, Università degli Studi di Milano, Milan, Italy

**Keywords:** systemic sclerosis, nailfold capillaroscopy, pulmonary arterial hypertension, echocardiography, right heart catheterization

## Abstract

Systemic sclerosis (SSc) is a connective tissue disease characterized by immune-system alterations, fibrosis involving the skin and internal organs and diffuse microangiopathy. Pulmonary arterial hypertension (PAH) is a severe complication of SSc affecting about 10–15% of the patients and it is a leading cause of mortality. Due to the devastating nature of SSc-PAH, there is a clear need to systematically adopt appropriate screening programs. Nail fold videocapillaroscopy (NVC) studies have shown a more severe peripheral microvascular dysfunction in SSc patients with PAH suggesting that abnormalities in peripheral microcirculation may correlate with pulmonary microangiopathy. This is a cross-sectional study involving four tertiary University Rheumatology Units in the Center-North of Italy. Seventy patients, 35 adults with SSc and PAH confirmed by RHC (F/M 34/1; median age 65.2 ± 8.9 SD yrs), and 35 SSc patients without PAH were enrolled (F/M 3471; median age 63.3 ± 10.3 SD yrs). Clinical, laboratoristic and instrumental data were collected and NVC was performed in all patient. Specific NVC parameters were evaluated and a semi-quantitative rating scale was adopted to score these changes. Finally, patients were distributed into the suitable NVC pattern belonging to the scleroderma pattern. Our aim was to compare the peripheral microangiopathy changes in SSc patients with and without PAH, and to investigate the relationship between NVC findings and the main hemodynamic parameters of pulmonary vasculopathy. Patients with SSc-PAH+ showed a significant higher frequency of interstitial lung disease (ILD). No significant differences regarding clinical and laboratoristic parameters were observed. NVC abnormalities, avascular areas were more frequent in SSc patients with PAH, respect to those without (*p* = 0.03), and capillary density was significantly lower when considering grade 3 (*p* = 0.02). A higher NVC semiquantitative mean was found in SSc-PAH+ patients and a greater rate of the “late” pattern was detected in SSc-PAH+ subjects in respect to PAH- (57.1% vs. 25.7%) (*p* = 0.03). A significant correlations between pulmonary pressure values (sPAP by TTE and mPAP by RHC) and the capillary density (Spearman's rho 0.35, *p* = 0.04 for both). Our findings provide additional evidence to the literature data, confirming that a higher degree of peripheral nailfold microangiopathy is more common in SSc-PAH patients, and further strengthening the concept that NVC changes may run parallel with similar abnormalities inside pulmonary microcirculation.

## Introduction

Pulmonary arterial hypertension (PAH) is a life-threatening and progressive disease characterized by vasoconstriction and remodeling of the pulmonary vasculature leading to increased pulmonary vascular resistance (PVR) that may result in right heart failure and death (1, 2).

According to the 6th World Symposium on Pulmonary Hypertension Task Force and the current Guidelines of the European Society of Cardiology/European Respiratory Society (ESC-ERS) (3, 4). PAH is defined by an elevated mean pulmonary arterial pressure (mPAP) >20 mmHg, normal pulmonary artery wedge pressure (PAWP) ≤ 15 mmHg, and elevated pulmonary vascular resistance (PVR) ≥3 Wood Units at rest. Some of the pathological changes involved with PAH are pulmonary endothelial dysfunction and inflammation promoting the remodeling of small- and medium-sized pulmonary arterioles thrombosis and obstruction of pulmonary blood vessels with proliferation of the vascular endothelium that may lead to the formation of the obstructive plexiform lesions (5). Recently, the possibility of a co-existing peripheral microangiopathy has been reported in idiopathic PAH (6, 7) and the peripheral microvascular changes play a decisive role also in systemic sclerosis (SSc). SSc is a challenging immune-mediated connective tissue disease (CTD) affecting skin and internal organs (8–11). SSc represents the main CTD associated with PAH occurring in approximately 10–15% of SSc patients (12–15). Despite the possibility of having new-targeted therapies, slowing down the progression of PAH, this condition is still a leading cause of death in SSc (16, 17). Therefore, an early accurate diagnosis should be mandatory to improve the survival of SSc patients (18). Nailfold video-capillaroscopy (NVC) is a well-known, validated, non-invasive imaging technique, which allows assessing peripheral microcirculation and diagnosing different diseases affecting peripheral microcirculation (19). Some NVC studies have shown a more severe peripheral microvascular dysfunction in SSc patients with PAH compared to those without PAH, suggesting that abnormalities in peripheral microcirculation may correlate with pulmonary microangiopathy (7, 20–24).

The aim of the present study was to compare the peripheral microangiopathy changes in SSc patients with and without PAH, and to investigate the relationship between NVC findings and the main hemodynamic parameters of pulmonary vasculopathy.

## Patients and Methods

This was a cross-sectional, case-control study involving four tertiary University Rheumatology Units in the Center-North of Italy with expertise in SSc diagnosis and management, as well as in NVC (19–21, 25, 26). PAH assessment was made by the local Cardiology Units with experience in right heart catheterization (RHC). All patients satisfied the ACR/EULAR 2013 classification criteria for SSc (27).

The DETECT-PAH algorithm (17, 18) was used to screen SSc patients and identify those with a high-risk of PAH. Briefly, the DETECT algorithm is a tool to identify patients with PAH in the asymptomatic stages, through the study of clinical variables, pulmonary function tests, immunological, biological, electrocardiographic and finally echocardiographic parameters. Those with a high PAH probability underwent RHC. On the contrary, RHC was not performed in those with a low probability of PAH due to ethical reasons.

Patients with SSc were divided into “cases,” those with a high probability of PAH by DETECT-PAH algorithm and a RHC-confirmed diagnosis of PAH (mPAP>20 mmHg+PAWP ≤ 15 mmHg + PVR≥3 Wood Units at rest) (3, 4) and “controls,” those with a low probability of PAH by DETECT-PAH algorithm. Controls were matched for sex, age, and disease duration.

Written informed consent was obtained from all participants and data were collected in a general database. The study received approval from the local Ethical Committees and performed according to the Declaration of Helsinki.

The patients' demographic and clinical findings were carefully considered. Data collected at registration included: age, disease duration, type of skin subset (limited/diffuse), presence of Raynaud's phenomenon (RP), modified Rodnan skin score, other skin involvement (subcutaneous calcinosis, telangiectasia), digital ulcers-DUs, lung involvement, gastro-intestinal symptoms (dysphagia, reflux), cardiopulmonary signs and symptoms (heart failure, pericardial effusion, dilated cardiomyopathy), sicca syndrome (xerostomia/xeropthalmia), and joint involvement (tenosynovitis, arthritis, tendon friction rubs), as previously described (20, 21, 28). Laboratory and instrumental evaluations included antinuclear antibodies (ANA), anti-extractable nuclear antigens (anti-ENA), SSc-related antibodies (mainly anti-centromere/CENP-B and anti-topoisomerase I/Scl-70), diffusion capacity for carbon monoxide (DLCo) and high-resolution computed tomography-HRCT were reported (17, 18, 20, 21, 28).

### Nailfold Videocapillaroscopy and Image Analysis

NVC was performed in all patients during their regular assessment (within 3-months before and after the RHC) using a videocapillaroscope with a 200x magnification optical contact probe. All fingers of both hands, excluding thumbs, were examined for each patient. Two adjacent fields of 1 mm in the middle of the nailfold were captured from all fingers at least, according to the current method (21, 25, 26). The derived digital images were stored and the same experienced investigator for each Unit (FI, DG, RDA, VR), blinded to the clinical data, was responsible for reviewing and scoring the NVC images.

The following parameters were considered, according to previous categorizing methods: presence of enlarged/giant capillaries, micro-hemorrhages, loss of capillaries (avascularity), disorganization of the vascular bed, morphology (tortuous, ramified/bushy capillaries, bizarre loops). Altered capillary flow, appearing as granular/sludge and loops' length (normal/short/elongated loops), were evaluated.

A semi-quantitative rating scale was adopted to score these changes: grade 0 = no changes; 1 = < 33%; 2 = 33–66% 3 = >66% of changes on the total number of capillaries/mm. The mean score for each subject was obtained from the analysis of all fingers assessed (19, 26).

The degree of capillary density was considered to be 0 when capillaries were >9/mm, 1 for 7–9 capillaries/mm, 2 for 4–6 capillaries/mm and 3 for <4 capillaries/mm.

The rating system for avascular areas (avascularity of the capillary bed) was classified as follows: grade 0 = no obvious avascular areas; grade 1 = mild (one or two discrete areas of vascular deletion); grade 2 = moderate (more than two discrete areas of vascular deletion); grade 3 = severe (presence of large, confluent avascular areas). Finally, patients were distributed into the suitable NVC pattern belonging to the scleroderma pattern: (i) early (few giant capillaries, few hemorrhages, relatively preserved capillary distribution, not obvious loss of capillaries). (ii) active (frequent giant capillaries, frequent hemorrhages, moderate loss of capillaries with some avascular areas, mild disorganization of the capillary bed, absent or some ramified capillaries). (iii) late (irregular enlargement of the capillaries, few or absent giant capillaries, absence of hemorrhages, severe loss of capillaries with confluent avascular areas, severe disorganization of the capillary array, frequent ramified/bushy capillaries) (19).

### Statistical Analysis

Qualitative variables (e.g., sex, clinical phenotype, organ involvement, laboratory data, medication use, and NVC findings) were reported using the absolute frequency and/or its corresponding percentage. Quantitative variables (e.g., age, diseases duration, echocardiographic and hemodynamic data) were reported using the mean ± the standard deviation (SD).

Baseline demographic, laboratory, and disease-related data were compared among cases and controls using the Chi-Square test (for qualitative variables) and Mann-Whitney U test (for quantitative variables). NVC findings were compared among those with and without a RHC-confirmed diagnosis of PAH using the Cochran-Armitage test for trend.

The correlation between hemodynamic and NVC findings was evaluated using the Spearman's rank correlation coefficient. A *p* value < 0.05 was considered significant. The analyses were carried out using STATA v.14.

## Results

70 patients, 35 adults with SSc and PAH confirmed by RHC, and 35 disease controls, matched for sex, age, and disease duration were enrolled in the study. Demographic, clinical, laboratory and hemodynamic parameters of SSc patients, with and without PAH, are given in Table 1.

**Table 1:** Demographic, clinical, laboratory and hemodynamic parameters of SSc patients, with and without pulmonary hypertension.

		**SSc PAH+ (*n* = 35)**	**SSc PAH- (*n* = 35)**	***p*-value**
	Sex (F. %)	34 (97.1%)	34 (97.1%)	0.99
	Age (years. mean ± SD	65.2 ± 8.9	63.3 ± 10.3	0.38
	Disease duration (months)	166.7 ± 121.3	168.3 ± 13.4	0.61
Clinical phenotype	Limited	28 (80.0%)	24 (68.6%)	0.27
	Diffuse	7 (20.0%)	11 (31.4%)	
Organ involvement	Raynaud's phenomenon	35 (100.0%)	35 (100.0%)	0.99
	Interstitial lung disease	20 (57.1%)	11 (31.4%)	0.03*
	Digital ulcers	9 (25.7%)	18 (51.4%)	0.03*
	Joint involvement	8 (22.9%)	12 (34.3%)	0.29
	Teleangectasias	24 (68.6%)	19 (54.3%)	0.22
	Subcutaneous calcinosis	8 (22.9%)	7 (20.0%)	0.77
	Xerostomia	12 (34.3%)	15 (42.9%)	0.46
	Xerophtalmia	11 (31.4%)	16 (45.7%)	0.22
	Gastrointestinal involvement	23 (65.7%)	25 (71.4%)	0.61
	Cardiac involvement	17 (48.6%)	12 (34.3%)	0.23
	Skin Score	8.7 ± 9.4	7.5 ± 5.2	0.39
Treatments	PGAs	12 (34.3%)	16 (45.7%)	0.33
	ERAs	23 (65.7%)	12 (34.3%)	0.01
	PDE5Is	14 (40.0%)	7 (20.0%)	0.07
Laboratory data	Antinuclear antibodies	29 (82.9%)	35 (100.0%)	0.01*
	Anti-Scl70	8 (22.9%)	14 (40.0%)	0.12
	Anti-centromere	17 (48.6%)	17 (48.6%)	0.99
Echocardiographic and hemodynamic findings	Right atrial area	24.6 ± 4.6	/	/
	Tricuspid regurgitation gradient	49.9 ± 13.1	/	/
	sPAP	59.9 ± 18.3	/	/
	mPAP	36.0 ± 9.4	/	/
	PAWP	10.4 ± 4.1	/	/
	TPR (Wood units)	8.6 ± 4.7	/	/

*PGAs, Prostacyclins and prostaglandins*.

*ERAs, Endothelin receptor antagonists*.

*PDE5Is, Phosphodiesterase inhibitors*.

*sPAP, Systolic pulmonary arterial pressure*.

*mPAP, Mean pulmonary arterial pressure*.

*PAWP, Pulmonary Artery Wedge Pressure*.

*TPR, Total Pulmonary Resistance*.

*p < 0.05.

**Figure 1:**
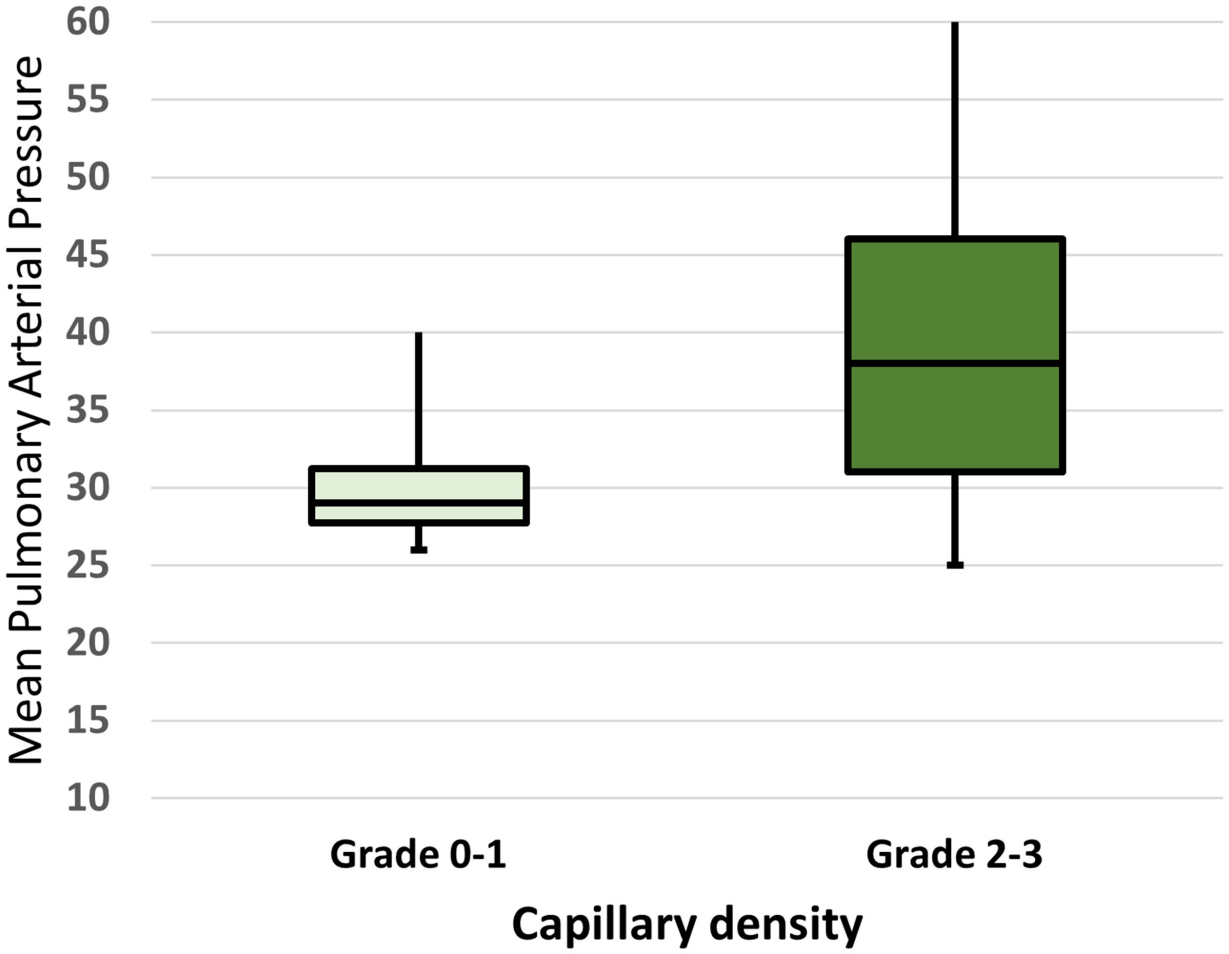
Mean pulmonary arterial pressure (mPAP) in SSc patients with a capillary density score of 0–1 vs. 2–3. The horizontal line in the box represents the median, lower and upper boundaries of the box represent the 25th (Q1) and the 75th (Q3) percentiles. Whiskers represent the minimum and the maximum value. The minimum, 25th, 50th (median) and 75th percentile and maximum values were 26.0, 27.5, 29.0, 31.5, and 48.5 in those with capillary density score of 0–1. The minimum, 25th, 50th (median) and 75th percentile and maximum values were 25.0, 31.0, 38.0, 46.0, 60.0 in those with capillary density score of 2–3. *P*-value for comparison: 0.01.

As regards NVC abnormalities, avascular areas were more frequent in SSc patients with PAH, respect to those without (*p* = 0.03), and capillary density was significantly lower when considering grade 3 (*p* = 0.02). Moreover, a higher NVC semiquantitative mean score (percentage of all abnormalities >66%) was found in SSc-PAH+ patients (Table 2). Finally, a greater rate of the “late” pattern was detected in SSc-PAH+ subjects in respect to PAH- (57.1% vs. 25.7%) (*p* = 0.03). When comparing the hemodynamic parameters in the group with PAH, we found significant correlations between pulmonary pressure values (mPAP by RHC) and the capillary density (Spearman's rho 0.35, *p* = 0.04 for both) (Table 3). No correlation between other abnormalities was detected, particularly regarding avascular areas (Table 3). Figure 1 highlights the correlations between mPAP by RHC along with the capillary density scores, knowing that scores 0–1 and 2–3 have been paired to better illustrate the statistical difference.

**Table 2:** Capillaroscopic findings in patients with or without pulmonary hypertension (PAH).

**PAH+** **(n°35) PAH- (n°35)**									
	**Grade 0**	**Grade 1**	**Grade 2**	**Grade 3**	**Grade 0**	**Grade 1**	**Grade 2**	**Grade 3**	***P-value***
Avascular areas	4 (11.4%)	7 (20.0%)	12 (34.3%)	12 (34.3%)	12 (34.3%)	11 (31.4%)	6 (17.1%)	6 (17.1%)	<0.01*
Loops' morphology	-	6 (17.1%)	13 (37.1%)	16 (45.7%)	-	14 (40.0%)	13 (37.1%)	8 (22.9%)	0.01
Capillary density	1 (2.9%)	3 (8.8%)	19 (55.9%)	11 (32.4%)	5 (14.3%)	9 (25.7%)	18 (51.4%)	3 (8.6%)	<0.01*
Loops' length	-	17 (48.6%)	18 (51.4%)	-	1 (2.9%)	17 (48.6%)	17 (48.6%)	-	0.63
Loops' dilatation	3 (8.6%)	11 (31.4%)	21 (60.0%)	-	6 (17.1%)	14 (40.0%)	14 (40.0%)	1 (2.9%)	0.19
Microhaemorrhages	9 (25.7%)	19 (54.3%)	7 (20.0%)	-	9 (25.7%)	11 (31.4%)	13 (31.7%)	5 (12.2%)	0.57
Loops' distribution	1 (2.9%)	2 (5.7%)	23 (65.7%)	9 (25.7%)	3 (8.6%)	4 (11.4%)	13 (37.1%)	11 (31.4%)	0.57
Capillary flow	1 (2.9%)	6 (17.1%)	16 (45.7%)	12 (34.3%)	-	11 (31.4%)	13 (37.1%)	11 (31.4%)	0.54
Mean NVC score	3 (8.6%)	17 (48.6%)	15 (42.9%)	-	9 (25.7%)	22 (62.9%)	4 (11.4%)	-	<0.01*
Scleroderma pattern	*NS*	*Early*	*Active*	*Late*	*NS*	*Early*	*Active*	*Late*	
	-	5 (14.3%)	10 (28.6%)	20 (57.1)	2 (5.7%)	12 (34.3%)	9 (25.7%)		<0.01*

*NVC, Nailfold Videocapillary*.

*NS, Non specific pattern*.

*P < 0.05*.

**Table 3:** Correlation between capillaroscopic parameters and the main haemodynamic/echocardiographic findings regarding 35 SSc patients with pulmonary hypertension.

	**mPAP**		**sPAP**		**Wood score**		**PAWP**	
**Scleroderma pattern**	**Rho** **0.08**	**p** **0.66**	**Rho** **0.11**	**p** **0.56**	**Rho** **0.14**	**p** **0.41**	**Rho** **0.16**	**P** **0.36**
Loop length	0.09	0.63	0.16	0.38	0.14	0.43	0.30	0.08
Architectural distribution	0.00	0.99	0.10	0.60	0.07	0.68	0.23	0.18
Capillary density	0.35	0.04*	0.35	0.04*	0.14	0.42	0.10	0.56
Loops' morphology	0.15	0.48	0.29	0.11	0.14	0.43	0.06	0.75
Loops' dilatation	0.10	0.56	0.04	0.81	0.18	0.29	0.02	0.91
Avascular areas	0.03	0.85	0.10	0.60	0.15	0.38	0.07	0.67
Microhaemorrhages	0.04	0.84	0.09	0.73	0.05	0.78	0.20	0.06
Capillary flow	0.08	0.66	0.20	0.27	0.07	0.70	0.15	0.37
Mean NVC score	0.20	0.26	0.23	0.21	0.01	0.94	0.04	0.83

*sPAP, Systolic pulmonary arterial pressure*.

*mPAP, Mean pulmonary arterial pressure*.

*PAWP, Pulmonary arterial wedge pressure*.

*NVC, Nailfold videocapillary*.

*p < 0.05.

## Discussion

The main result of our study was that a higher degree of peripheral nailfold microangiopathy, mainly avascular areas and low capillary density is more common in SSc-PAH patients.

Microvascular dysfunction plays a key role in SSc pathogenesis, leading to the typical clinical manifestations of the disease, such as RP, DUs, skin and internal organs involvement (9–11). Furthermore, endothelial disfunction and vascular inflammation in SSc result in accelerated atherosclerosis and macrovascular damage, as demonstrated by Ciccone et al. (29), which observed an increased carotid intima-media thickness values in SSc compared to NoSSc patients and controls.

Hearth is frequently affected, and the burden of cardiac complications leads to a reduction in life expectancy of these patients (12–15). In particular PAH is characterized by increased resistance of pulmonary vessels because of remodeling and obstruction of pulmonary arterioles with subsequent increase of the mean pulmonary artery pressure and is usually diagnosed 10 to 15 years after the onset of the disease, so the majority of the SSc patients are usually presented with serious manifestations, severe hemodynamic impairment and associated with poor prognosis and increased mortality especially in male subjects (18, 28, 30). Early screening through systematic evaluation of asymptomatic SSc patients could diagnose PAH at an early stage with a consequent better prognosis of the disease (17).

NVC is a safe, inexpensive, simple, and non-invasive imaging technique used to analyze the morphology of capillaries mainly in the nailfold area (19), providing a potential early screening tool for the diagnosis of otherwise asymptomatic organ involvement, such as PAH (31, 32).

Previous studies demonstrated some correlations between NVC abnormalities and the severity of internal organs involvement, including PAH (20, 23, 24, 30, 31). In a 3-year prospective study, the sequential loss of capillaries was recognized as a marker for the occurrence of PAH (31). SSc-PAH patients have also been associated with higher scores of capillary loss and disorganization of the nailfold capillary bed (32) and among observational studies, that employed RHC for PAH diagnosis, capillary density was found significantly reduced in SSc-PAH+ patients (21, 22, 32). In our study, the largest group of SSc-PAH+ patients so far investigated, we observed a significant extremely low degree of capillary density (<4 loops/mm) in PAH-SSc patients, in agreement with the finding of the previous studies. Our data confirmed the higher NVC rating scores more frequent in the SSc using the semi-quantitative assessment, in SSc-PAH group (Table 2) while, using the same scoring method for avascular area, we reported the higher frequency of capillary dropout in SSc-PAH+ patients (21) as reported in the literature studies (21, 22, 32). We also evaluated the different qualitative patterns across studies (21–24, 32). Corrado et al. (22) found that the percentage of patients presenting the more severe NVC patterns (active/late) was overall significantly greater in SSc-PAH+ compared to SSc-PAH- (73.2% vs. 50% respectively. Hofstee et al. (33) reported a lower capillary density in SSc with PAH, although loop dimensions were comparable. Finally, Riccieri et al. described more severe NVC patterns (active/late) in 11 (92%) and only in 5 (42%) patients, respectively (21). Our data are almost overlapping, confirming a higher significant percentage of the active/late pattern in our SSc-PAH+ patients (*p* = 0.03) (Table 2). It should be emphasized that both “active” and “late” patterns are characterized by the presence of discrete/large areas of capillary loss (19), reflecting a greater internal organs involvement in respect to the presence of the “early” pattern (34).

Our study confirmed even the relationship between echo and/or RHC detected mPAP, and capillary density, in particular the increase of PAP was related to the decrease of the number of nailfold capillaries. Preliminary data reported that increasing echocardiographically estimated sPAP correlates with the severity of the scleroderma pattern (34), particularly with the late pattern. Our data more consistently support the idea that a lower capillary density of the peripheral microcirculation reflects increased pressures at the pulmonary artery level (35). The NVC capillaries changes might reveal what is going on in the pulmonary circulation, supporting the possible hypothesis that nailfold microangiopathy may be related to those vascular abnormalities presenting in the pulmonary circulation with reduced capillary density and broad avascular areas. Another valuable observation of the study was the female prevalence in our PAH patients' cohort, confirming the registries worldwide PAH data showing a female predominance of pulmonary hypertension. Dysregulation of estrogen synthesis and metabolism seems to play a major role in these sex-related differences (28, 35), so further analyses on larger sample size are needed to better understand the penetrance of PAH in SSc women in order to translate to a better prognosis and/or a better quality of life. Finally, if the association between PAH and ILD is expected in SSc (17, 18), even together with NVC alterations (36), the lack of association with DU's is conflicting and needs of further investigation (37).

The study has also a few limitation. Although this was the largest multicentric study on this topic so far, the sample size is relatively small and it does not allow us to take into account other potential confounders. Second, a formal reliability exercise among cardiologists performing echocardiographic examinations was not carried out before the study's start. However, the participating centers have a great experience in the diagnosis and management of SSc and followed shared procedural protocols.

In conclusion, we found precise NVC changes using both a specific evaluation system of avascular areas and capillary density, as well as a semi-quantitative evaluation scale of overall scores. More specifically, a clear association emerged between scores referring to low capillary density/avascular areas and the presence of PAH, also with respect to the qualitative assessment, through the scleroderma pattern late. In addition, low capillary density correlates directly with mean pulmonary pressure. Overall, our findings provide additional evidence to the literature data, confirming that a higher degree of peripheral nailfold microangiopathy is more common in SSc-PAH patients, and further strengthening the concept that NVC changes may run parallel with similar abnormalities inside pulmonary microcirculation (23, 24). If confirmed by further investigations in larger patients' series, NVC microvascular alterations could be included in the armamentarium of PAH early detection and so to contribute to a better survival of the patients. There is a need for prospective, multicenter, possibly cross-national studies to validate capillaroscopic findings in the early recognition of this life-threatening condition.

## Data Availability Statement

The original contributions presented in the study are included in the article/supplementary material, further inquiries can be directed to the corresponding author.

## Ethics Statement

The studies involving human participants were reviewed and approved by Local Ethical Committees. The patients/participants provided their written informed consent to participate in this study.

## Author Contributions

All authors listed have made a substantial, direct, and intellectual contribution to the work and approved it for publication.

## Conflict of Interest

The authors declare that the research was conducted in the absence of any commercial or financial relationships that could be construed as a potential conflict of interest.

## Publisher's Note

All claims expressed in this article are solely those of the authors and do not necessarily represent those of their affiliated organizations, or those of the publisher, the editors and the reviewers. Any product that may be evaluated in this article, or claim that may be made by its manufacturer, is not guaranteed or endorsed by the publisher.
